# The Influence of Environmental Exposure to Xenoestrogens on the Risk of Cancer Development

**DOI:** 10.3390/ijms252212363

**Published:** 2024-11-18

**Authors:** Martyna Gachowska, Alicja Dąbrowska, Bartosz Wilczyński, Jacek Kuźnicki, Natalia Sauer, Wojciech Szlasa, Christopher Kobierzycki, Zofia Łapińska, Julita Kulbacka

**Affiliations:** 1 Faculty of Medicine, Wroclaw Medical University, Pasteura 1, 50-367 Wroclaw, Poland; martyna.gachowska@student.umw.edu.pl (M.G.); alicja.dabrowska@student.umw.edu.pl (A.D.); bartosz.wilczynski@student.umw.edu.pl (B.W.); jacek.kuznicki@student.umw.edu.pl (J.K.); 2 Department of Clinical Pharmacology, Faculty of Pharmacy, Wroclaw Medical University, Borowska 211A, 50-556 Wroclaw, Poland; natalia.sauer@umw.edu.pl; 3 Department of Molecular and Cellular Biology, Faculty of Pharmacy, Wroclaw Medical University, Borowska 211A, 50-556 Wroclaw, Poland; wojciech.szlasa@umw.edu.pl; 4 Division of Histology and Embryology, Department of Human Morphology and Embryology, Wroclaw Medical University, 50-368 Wroclaw, Poland; christopher.kobierzycki@umw.edu.pl; 5 Department of Immunology and Bioelectrochemistry, State Research Institute Centre for Innovative Medicine, LT-08406 Vilnius, Lithuania

**Keywords:** xenoestrogens, estrogens, isoflavones, pesticides, environmental exposure

## Abstract

Xenoestrogens (XEs) are a group of exogenous substances that may interfere with the functioning of the endocrine system. They may mimic the function of estrogens, and their sources are plants, water or dust, plastic, chemical agents, and some drugs. Thus, people are highly exposed to their actions. Together with the development of industry, the number of XEs in our environment increases. They interact directly with estrogen receptors, disrupting the transmission of cellular signals. It is proven that XEs exhibit clinical application in e.g., menopause hormone therapy, but some studies observed that intense exposure to XEs leads to the progression of various cancers. Moreover, these substances exhibit the ability to cross the placental barrier, therefore, prenatal exposure may disturb fetus development. Due to the wide range of effects resulting from the biological activity of these substances, there is a need for this knowledge to be systematized. This review aims to comprehensively assess the environmental sources of XEs and their role in increasing cancer risk, focusing on current evidence of their biological and pathological impacts.

## 1. Introduction

The estrogens are a large group of sex steroid hormones produced by the endocrine system, playing a significant role in both male and female organisms. In addition to controlling female reproductive function and the development of secondary sexual characteristics, estrogens are responsible for regulating glucose and lipid levels, bone metabolism, skeletal growth, brain functioning, and the control of inflammation, which is essential for the maintenance of homeostasis in human bodies [1,2]. According to the available literature, four main estrogens may be distinguished: estrone (E1), estradiol (E2), estriol (E3), and estetrol (E4—only present in pregnancy from 9 weeks of gestation, with its physiological role remaining unknown [3]. Each of these substances exhibits a steroid-like structure, and therefore diffuses across the cell and nuclear membrane to interact with receptors. There are two main estrogen receptors, alpha and beta (ERα and ERβ, respectively)—both members of the steroid/nuclear receptor superfamily. Studies have shown that ERβ is predominantly present in the ovaries, testes, prostate gland, bladder, urethra, lungs, and the circulatory system. In contrast, ERα is primarily found in the immune system, the male reproductive system, and adipocytes. Both are also present in the central nervous system [4]. ER may be stimulated by two classic mechanisms: (1) “direct binding” of hormones and, (2) ‘tethering’ which requires coactivator components. Upon the binding of estrogen, the ER undergoes a conformational change that allows it to dimerize and translocate to the nucleus, where it regulates gene expression [5].

Nowadays, our environment is rich in hormone-like agents that can mimic the action of endogenous estrogens. Xenoestrogens (XEs) are defined as endocrine-disrupting chemicals (EDCs), which may disrupt the normal function of the endocrine system [4,6]. Classical estrogens and XEs share several similarities that influence their interactions with the body, particularly through their receptors. XEs can also bind to the ER, but the affinity and potency of these compounds vary widely [7]. Some XEs, such as genistein, have a higher affinity for the ER than endogenous estrogen, while others, such as p-nonylphenol, have a lower affinity.

**Figure 1:**
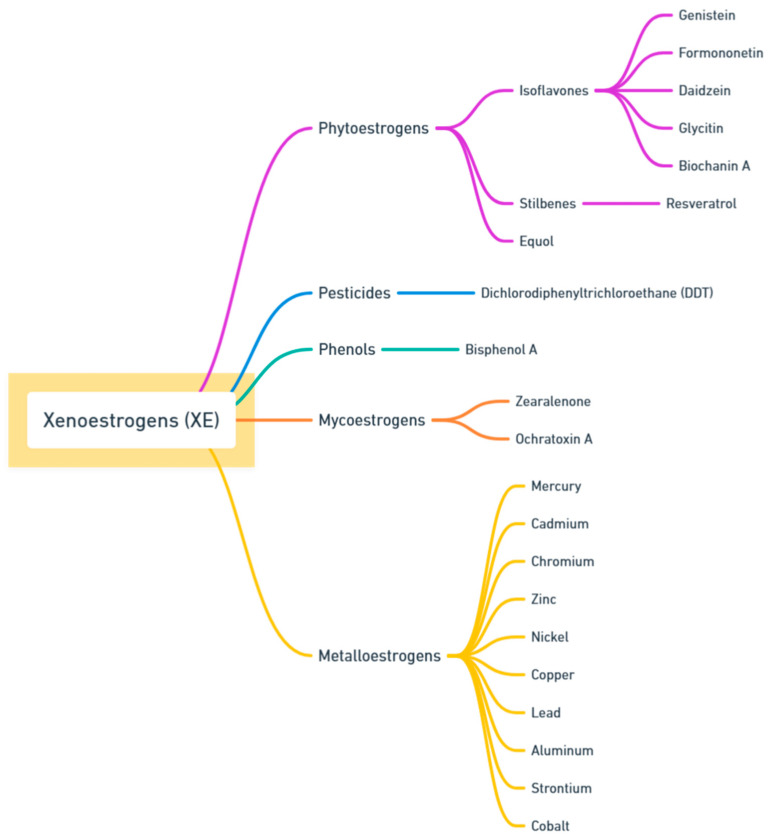
Representative classification of various types of xenoestrogens (XEs), including phytoestrogens, pesticides, phenols, mycoestrogens, and metalloestrogens. Each category divisions into specific compounds, highlighting the diversity of XEs sources and their potential impact on biological systems (created by https://whimsical.com/, accessed on 6 November 2024).

Unlike estrogens, XEs cannot be metabolized properly and may remain in the body, affecting hormone signaling [8]. Their actions are unpredictable, functioning as either estrogen mimics that activate receptor pathways or as anti-estrogens that impede normal estrogenic activity. The ability to bind to ERs due to the similar chemical structure means that XEs may act as estrogen agonists or antagonists [9]. In this study, we focused on the most prevalent XEs encountered in everyday life. This heterogeneous group includes phytoestrogens (stilbenes as resveratrol and isoflavones as genistin, daidzein, glycitin, biochanin A, formononetin, equol); pesticides (dichlorodiphenyltrichloroethane); phenols (bisphenol A); methaloestrogens (cadmium and aluminum, zinc, copper, cobalt, nickel, lead, mercury, strontium, chromium); and mycoestrogenes (zearalenone and ochratoxin A). The representative classification of XEs is presented in Figure 1. The XEs are to be found in food products, sealants, plastics, cigarette smoke, cosmetics, and detergents, as well as in paints or varnish—consequently, people are highly exposed to their actions. Numerous investigations propose that heightened exposure to XEs during fetal and neonatal stages can potentially impact fetal growth, instigate anomalies in testicular development, and result in modifications to male fertility in adulthood [10,11,12,13]. Interestingly, the dualistic nature of XEs as both agonists and antagonists of estrogen receptors leads to complex biological outcomes. Some XEs have been shown to promote tumor growth, while others inhibit cancer progression [14]. The alterations in gene expression depend on the exposure time and intensity to chemicals.

XEs can also interact with GPERs (G protein-coupled estrogen receptors). These receptors are involved in a variety of signaling pathways, including those that regulate hormone secretion, neurotransmitter release, and immune cell activation. Some XEs, such as bisphenol A (BPA), have been shown to activate GPERs and elicit downstream effects in various tissues [15]. XEs can also exert their effects via non-genomic signaling pathways and epigenetic modifications [16,17]. Non-genomic signaling pathways involve rapid, non-transcriptional responses to estrogen signaling, including activation of protein kinases and calcium signaling. Epigenetic modifications involve alterations to gene expression that do not involve changes to the DNA sequence itself, such as DNA methylation and histone modification. A better understanding of the mechanisms by which XEs act via transmembrane receptors is crucial for developing effective strategies to mitigate their health risks. This includes identifying which receptors XEs bind to and how these compounds alter downstream signaling pathways. Additionally, understanding the effects of XEs on non-genomic signaling pathways and epigenetic modifications may provide new insights into the mechanisms by which these compounds disrupt normal cellular function. Furthermore, research on the mechanism of xenoestrogen action via estrogen-related receptor gamma (ERRγ) has been ongoing for several years. The available studies have utilized various techniques, including cell-based assays, animal models, and structural biology, to elucidate the molecular details of this interaction. In vitro studies have been used to assess the ability of XEs to activate ERRγ and induce downstream gene expression [18]. Zhang et al. revealed that BPA may increase the invasion of triple-negative breast cancer (TNBC) cells [19]. Moreover, an in silico study demonstrates that the di(2-ethylhexyl)phthalate (DEHP) could activate ERRγ [20]. These studies and others have provided evidence that XEs can interact with ERRγ and induce transcriptional activity.

Animal models have also been used to study the effects of XEs on ERRγ signaling. For example, one study showed that exposure to BPA could induce ERRγ expression in the mammary gland of rats, suggesting that ERRγ may play a role in the development of breast cancer [21,22,23]. Overall, these studies and others have provided valuable insights into the mechanisms of xenoestrogen action via ERRγ. Further research is needed to fully understand the molecular details of this interaction and its implications for human health.

## 2. Methodology

This paper presents a study on environmental xenoestrogen exposure and its influence on cancer development. As those substances surround us, summarizing the available findings regarding this topic can serve to both gather the existing knowledge and indicate what can be further researched. To ensure a well-balanced review process and reduce biases, a clear review question was formulated (what are the most common XEs in our daily life and what is their potential impact on our health), a protocol was developed by implementing inclusion and exclusion criteria, a detailed literature search was performed, and finally, all the gathered information was summarized. The PubMed database was used as a main literature resource. The inclusion criteria included the accessibility of the resources and the publication types, such as case studies, original studies, experimental studies and WHO Reports. Studies carried out after 1999 were the main focus. However, earlier writings was not excluded if it contained up-to-date information. The exclusion criteria consisted of non-English publications or the ones without approved ethics notifications. Studies in which the outcomes were self-reported rather than objectively measured were excluded. Studies regarding substances other than XEs were not used.

## 3. The Most Common XEs

### 3.1. Phytoestrogenes

Phytoestrogens can be divided into isoflavones and stilbenes. Stilbenes such as resveratrol and isoflavone phytoestrogens, which are the most estrogenic flavonoids, contain genistein (7,4′-dihydroxy-6-methoxyisoflavone), daidzein (7,4′-dihydroxy isoflavone), glycitin (7,4′-dihydroxy-6-methoxyisoflavone), biochanin A (5,7 dihydroxy-4′-methoxy isoflavone), and formononetin (7-hydroxy-4′-methoxyisoflavone) [4,24]. Sometimes equol is also classified as an isoflavone [25]. However, it is worth noting that it is a metabolite of daidzein produced by intestinal bacteria, which does not include equol as a phytoestrogen [26].

#### 3.1.1. Isoflavones

The active form of isoflavones—aglycones (activated by microbiota of small intestine) is structurally similar to 17β-estradiol (E2), due to the presence of a phenolic ring and the distance between the two opposing phenolic oxygens these aglycones possess the ability to bind with ER [25,26]. Isoflavones demonstrate a greater affinity for ERβ than ERα. On average, isoflavones exhibit 100–500 times smaller affinity to ER than E2, and only genistin has a comparable affinity to 17β-estradiol [4]. Isoflavones belong to selective estrogen receptor modulators (SERMs). Interestingly, they have both agonistic and antagonistic effects [27]. However, it is not yet certain whether isoflavones can displace E2 due to their ability to bind to a primary site on the ER (act as competitive inhibitors) or their ability to bind to the active site of the ER (act as a non-competitive inhibitor). Furthermore, it was proven that isoflavones suppress 5α-reductase—the enzyme responsible for the conversion of testosterone (T) into 5α-dihydrotestosterone—and aromatase (an enzyme responsible for the transformation of T into E2 activity) [28,29].

The primary origins of these compounds are leguminous plants belonging to the Fabaceae family [30]. The quantity of phytoestrogens within a plant is predominantly contingent upon specific species and environmental factors, encompassing temperature, precipitation, harvesting timeframe, soil fertility, and post-harvest handling [31]. Isoflavone concentrations exhibit augmentation under stressful conditions, such as low humidity or pathogenic assaults [26,32,33]. Predominant contributors to isoflavone content are soy-based products, boasting approximately 1.5 mg/g (1.2–4.2 mg/g dry weight), whereas soy derivatives typically exhibit lower concentrations [33]. Additional sources encompass chickpeas, beans, and, to a lesser extent, certain fruits, vegetables, and nuts [34]. Furthermore, isoflavones are present in dietary supplements to alleviate menopausal symptoms in women. For this purpose, they are extracted from red clover, which contains as much as 10–25 mg/g dry weight of phytoestrogens [35]. In Western populations (mainly American), the consumption of cow’s milk and dairy products has been found to contribute significantly to the body’s supply of isoflavones [36]. Monitoring their levels is important because of the endocrine-disrupting properties [37]. Isoflavones can potentially pose a risk to the fetus in the uterus and the infant fed with the mother’s milk [38]. However, a study by Testa et al. shows that only children with congenital hypothyroidism can have problems and require modulation of thyroid hormone replacement doses [39]. It should be noted that the serum concentrations of genistein in soy formula-fed infants are 10 to 100 times higher than in the general population, thus infants’ intestines are more exposed [40].

#### 3.1.2. Stilbenes

Resveratrol (2,4′,5 trihydroxystilbene) is the major active compound of stilbenes and is mainly found in plants. Spermatophytes synthesize it in response to an injury, e.g., fungal infection. From the ancient ages, it was used in traditional oriental medicine, which was presumed to be beneficial to human health [41]. It can be found in grape skin and thus in wine (pinot grigio, made from red wine, was much richer in resveratrol than white grape wine such as chardonnay). There are distinguished cis and trans isoforms; interestingly, only the trans form was identified in grapes. Due to the similarity of trans-resveratrol and synthetic estrogens, resveratrol in the concentration required for biological effect (3–10 µmol/L) can compete with binding to ER. Resveratrol possesses antioxidative properties and thus plays a significant role in the prevention of cardiovascular diseases. Furthermore, resveratrol exhibits anticancer properties. It induces the detoxification of carcinogens due to its antioxidative and antimutagenic properties, modulation of the inflammatory process (counteracting NF-k B and AP-1 transcription), its inhibition of cyclooxygenase-1 and hydroperoxidase and the fact that it induces cell differentiation, thereby contributing to the prevention of tumor initiation [42].

In other studies it was demonstrated that bone cancer pain (BCP) rats exhibit reduced activity of adenosine monophosphate-activated protein kinase (AMPK), increased activity of the mitochondrial fission-associated protein Drp1 GTPase, along with mitochondrial dysfunction and irregular expression of BAX and Bcl-2 in their spinal cord. Notably, these alterations were reversed by resveratrol. Studies have demonstrated that resveratrol supports mitochondrial function by activating AMP-activated protein kinase (AMPK) and decreasing Drp1 activity, which in turn helps prevent the reduction of mitochondrial membrane potential caused by tumor necrosis factor-α. Additionally, research on a rat model of lung cancer has shown the anti-cancer potential of resveratrol. In this study, resveratrol was administered alongside benzo[a]pyrene, where it exhibited protective effects against carcinogenesis in the lungs. It was found to influence treatment and anticancer properties, mainly by moderating the raised activity of aryl hydrocarbon hydroxylase [43].

Moreover, another study investigated the effects of trans-resveratrol on anxiety-like behaviors and fear memory deficits induced by the time-dependent sensitization (TDS) model in rats, which simulates post-traumatic stress disorder (PTSD). Trans-resveratrol (10, 20, and 40 mg/kg, via gavage) reversed TDS symptoms and normalized TDS-induced alterations in the limbic hypothalamus-pituitary-adrenal (L-HPA) axis by reducing adrenal gland size and corticotropin-releasing factor (CRF) levels, and restoring glucocorticoid receptor expression in the hypothalamus, hippocampus, and amygdala. Neurobiological analyses showed that trans-resveratrol increased phosphorylation of cAMP response element binding protein (pCREB) and levels of brain-derived neurotrophic factor (BDNF), both of which were decreased in TDS-exposed rats. These findings indicate that trans-resveratrol protects neurons from PTSD-like stress by regulating L-HPA axis function and activating neuroprotective pathways involving pCREB and BDNF [44].

Thus, resveratrol might be a potentially interesting tool for both cancer prevention and therapy by inhibiting angiogenesis through the suppression of VEGF and matrix metalloproteinases, as well as activating p53 to stimulate apoptosis and induce cell cycle arrest [4,45,46,47].

### 3.2. Pesticides

Pesticides are a large group of chemicals that, in various forms and concentrations, are used in agriculture, vector control, household, and sanitary bathing for animals. The lack of awareness of their side effects, poor agricultural practices, and improper removal of containers increases the risk of exposure [48]. Pesticides are the main chemicals used in agriculture to kill insects (insecticides), plants (herbicides), and fungi (fungicides) [49]. It is estimated that five billion pounds of pesticides are used in the world, including penetrating plants, products, and water. Moreover, this number is constantly growing, giving more and more evidence that their residues can easily penetrate plant products and water [50]. The high exposure to pesticides has the workers in agricultural fields and the pesticide industry [51]. In addition to agricultural use, it applies to the countryside, the timber industry, public hygiene, and the food service sector, which means that the professional groups that may potentially be exposed to pesticides include gardeners, employees of golf courses and other sports facilities, road maintenance workers, railroads, lines electricity, municipal employees, etc. [52]. Children are particularly vulnerable to exploratory behavior that increases exposure to pesticides present in the ground, the risk of unintended ingestion of pesticides stored within reach of children and non-original containers, interaction with animals that have been treated with pesticides, and contact with pesticide residues in gardens and playgrounds [48]. Anatomical changes occur during infancy and childhood due to the biochemical and physiological responses in children and adults differ significantly. These changes affect the absorption, distribution, metabolism, and elimination of chemicals; therefore, organs in infants and children are more susceptible to pesticide damage [53]. The study conducted by Salameh et al. revealed that some specific physiological characteristics, such as large skin surface and body mass ratio, increased the sensitivity of cholinergic receptors to pesticides in children [54]. In the general population, fruits and vegetables are the major routes of chronic pesticide exposure. The USDA Pesticide Data Program (PDP), which has been systematically examining pesticide residues in vegetables and fruit sold in the US since 1991, reports that over 50% of conventionally grown vegetables and fruits in the US contain detectable levels of pesticides, and 31% contain two or more pesticides [55]. More than one hundred pesticides with different modes of action have been classified as endocrine disruptors [56]. The best-known, historic pesticide with such properties is dichlorodiphenyltrichloroethane (commonly known as DDT) [57]. DDT is an example of a highly beneficial chemical that, on the one hand, has saved millions of lives and, on the other, has proved highly toxic, leading to a declining bird population [58]. Mostafalou et al. described that a huge amount of evidence shows that exposure to pesticides can affect human diseases such as cancer, Alzheimer’s, Parkinson’s, amyotrophic lateral sclerosis, asthma, bronchitis, infertility, birth defects, attention deficit hyperactivity disorder, autism, diabetes, and obesity [59]. Carcinogenicity is considered the most commonly reported toxicity studied for each group of pesticides [60].

### 3.3. Metaloestrogens

To date, only organic compounds characterized by phenolic or ring structures have been described as having estrogenic activity; however, the recently published literature reports that metal ions may also interfere with estrogen action. Considering this, the group of those molecules has been defined as metaloestrogens and includes cadmium, aluminum, zinc, copper, cobalt, nickel, lead, mercury, strontium, chromium, etc. Among the XEs discussed, cadmium (Cd) and aluminum (Al) are the most extensively documented, and this paper will primarily focus on these two [14]. However, it is important to note that other metals, such as zinc, cobalt, copper, nickel, lead, mercury, and chromium, can also bind to estrogen receptors (ERs). Cobalt and cadmium, in particular, can displace zinc from the “zinc finger” motifs in the DNA-binding domain (DBD) of ERs [61]. Denier et al. [62] demonstrated that cadmium, copper, and zinc potentiate the E2-induced response in a dose-dependent manner.

#### 3.3.1. Cadmium

Humans are exposed to Cd (cadmium) through the inhalation of cigarette smoke, working in industrial plants such as metallurgy and electroplating, and consuming Cd-contaminated food [63]. Cd has a high transfer rate from soil to plants, making it present in the daily diet [64]. The safe limit for Cd intake is 7 μg Cd/week/kg body weight (70 μg/day for a 70 kg person) and was based on a critical kidney Cd concentration of 100–200 μg/g wet weight, corresponding to a urinary concentration threshold of 5–10 μg/g creatinine [65,66]. However, various research groups have revealed that urinary Cd levels below 0.5 μg/g creatinine are also associated with negative effects on the kidneys [64,67]. Foods rich in Cd include bivalves and shellfish, as well as filtering and collecting metals from the aquatic environment regardless of environmental pollution, although polluted waters may further increase the content of this and other metals in them [68]. It has been shown that some Pacific oysters can contain Cd in the amount of 13.5 mg/kg dry weight, and some New Zealand oysters can have this value even twice as high [69]. McKenzie et al. investigated 57 men and 19 women aged 20–75 years, whom he divided into four groups depending on the number of oysters consumed per week. The estimated Cd consumption for individuals in groups 1, 2, 3, and 4 was 34, 75, 116, and 250 μg/day, respectively. However, the study found that, in group 4, the increase in blood Cd associated with oyster consumption was only 1.2 μg/L. This suggests that interactions with selenium and other metals in oysters may reduce Cd absorption. However, later studies suggest that Cd in oysters and crustaceans is bioavailable and that long-term consumption of oysters causes a greater burden on the body of Cd [63,69,70]. In the studies of Copes et al., it was found that a significant increase in blood and urine Cd levels with an average consumption of 18 oysters/week (87 g/week) was associated with an oyster farming period of at least 12 years [69]. In addition to shellfish, a high concentration of Cd is also found in offal, such as liver and kidneys, in oilseeds, cocoa beans, and some forest mushrooms [71,72]. Rice consumption, which grows in flooded fields rich in Cd, is also a source of Cd [73]. Drinking water intake points and rivers near factories or mines can also be sources of Cd pollution [74]. Cd may enter the body through smoking tobacco products [75] Cigarettes, depending on the type, contain 1–2 μg of Cd, and a person who smokes twenty cigarettes per day introduces about 1 μg of Cd in the body; smokers have a significant increase in Cd concentration in blood serum and urine [76]. Cd can also be a professional risk because it is used in electroplating, industrial paints, and fertilizers [72]. It is proven that Cd mimics the effect of E2 and exhibits a stronger affinity to ERα, with potent estrogenic activity. Cd binds to the hormone-binding domain of Erα, increases ERα-mediated cell proliferation, and induces mitogenesis [62,77,78].

Furthermore, Cd can displace the zinc ion from the zinc finger motifs of ER [79]. Thus, Cd modifies the ability of DBDs to bind to EREs and disrupts gene expression [80]. Cd, according to a study by Garcia-Morales et al., decreases the level of ER mRNA and increases transcription of the progesterone receptor (PR) gene [81]. Authors reported cadmium-induced proliferation of the human breast cancer cell line MCF7 in a dose-dependent manner and increases the transcription and also expression of the progesterone receptor, moreover activates ERα in transient transfection assays. A Cd concentration of both 10 and 100 nM is able to stimulate MCF7 cell line growth. The effect of Cd was blocked by the antiestrogens, which supports the suggestion that Cd mediates the ERα genomic pathway. It was also proven that Cd activates the nongenomic ERα pathway [82].

Cd was reported to affect organs such as the lungs, kidneys, and especially the prostate, breast, and endometrium, where it can develop steroid-dependent tumors, inducing pathways normally activated by estrogens [83,84]. There are four identified ways in which Cd could affect carcinogenesis: aberrant gene expression, inhibition of DNA repair, inhibition of apoptosis, and induction of oxidative stress [85]. Most significant are mechanisms targeting DNA repair processes, tumor suppressors, and signal transduction proteins, with all of them acting concurrently, increasing the risk of carcinogenesis [86]. Studies support that elevated Cd exposure and its elevated levels in the body are associated with increased breast cancer incidence [87]. Other epidemiological studies demonstrated that Cd exposure increases the risk of bladder cancer development and renal cancer in occupationally exposed populations, as well as ovarian cancer [84,85,88].

#### 3.3.2. Aluminum

Al (aluminum), following oxygen and silicon, is the third most common element on Earth, and due to its properties, it is used in many technical products and processes [89]. In 2017, approximately 64 million tons of Al were produced worldwide [90]. Food has been the most important factor causing oral exposure to this metal [91]. A German pilot total diet study (TDS) showed the presence of Al in 86% of the 243 samples. The most abundant Al was in the group: “legumes, nuts, oilseeds, spices”, which had an average Al content of 28.5 mg/kg, and the group “water-based sugars, sweets and sweet desserts” with an average Al content of 21, 1 mg/kg. For other food groups, the Al content ranged between 0.1 and 5.2 mg/kg, and in the group “animal and vegetable fats and oils”, no Al was detected at all [92]. It was also found that the average weekly exposure to Al in Germany from food intake is 50% of the Tolerable Weekly Intake (TWI) of 1 mg/kg body weight (BW)/week, as determined by the European Food Safety Authority (EFSA) [89]. Conversely, the average consumption of Al by adults in France was defined as 0.28 mg/kg BW/week and 0.49 mg/kg BW/week for consumers with high consumption [92]. In the Italian adult population, it was 4.1 mg/day (corresponding to 0.48 mg/kg BW/week; BW = 60 kg) [93]. Another, in terms of importance, source of exposure to Al is the use of cosmetics, especially antiperspirants, which, according to the estimated exposure, may lead to reaching or even exceeding the tolerable weekly intake [94].

AGES (Österreichische Agentur für Gesundheit und Ernährungssicherheit GmbH (2017) reported that antiperspirants contain 0.2–5.8% Al, which means that with an average consumption of 1.5 g of antiperspirant per day, the exposure for adults would be 0.69 mg/kg BW/week and 0.98 mg Al/kg BW/week for children aged 11 to 14 years [89,95]. Examination of the Al content of fourteen sunscreen samples showed an Al content of 0.1% and a maximum of 0.8% [92]. Assuming an average use of 18 g of sunscreen for 25 days a year [96], with 0.1% Al in the cream, the exposure for adults would be 0.02 mg Al/kg BW/week and 0.16 mg Al/kg BW/week with 0.8% Al content in the cream. The exposure for children aged 1, 5, and 10 years would be at the level of 0.26, 0.24, and 0.21 mg Al/kg BW/week, respectively. Conversely, whitening pastes may contain Al fluoride, and the tests showed the average Al content in the 15 tested products at the level of 0.9% with a median of 0.02% and the highest value of 3.9%. In line with the average paste consumption, this would lead to an exposure of 0.003 mg Al/kg BW/week, and for children 11 to 14 years of age, an exposure of 0.005 mg Al/kg BW/week [95]. Al can also enter the body by inhalation, but it has been found that, apart from areas close to intensive Al extraction, there is no significant inhalation exposure [97]. Al, following oxygen and silicone, is the third most common element on earth, and due to its properties, it is used in a wide range of technical products and processes [89]. It may be absorbed by human organisms directly through the skin. Additionally, Al may enter the body by inhalation, but it has been found that, apart from areas close to intensive Al extraction, there is no significant inhalation exposure [97]. Usually, aluminum enters organisms in the form of aluminum chloride or aluminum chlorohydrate. These compounds inhibit the binding of E2 to ER. However, the mechanism of inhibition is still not well determined. Based on the study by Darbre et al., there is no reason to assume that the mechanism of the competition involves binding to the LBD of ER [98]. Tsialtas et al. [99] demonstrated that aluminum in the form of aluminum chlorohydrate (ACH) reduces ERβ protein levels, which may be associated with an increase in ERα protein levels. According to Un-Gi Hwang Al, it interferes with the ER by depressing the upregulation of ER activity [93]. It is determined that Al can cause genomic instability that could lead to cancer development and increase migratory and invasive properties of the cell necessary for metastasis. It is also suggested that exposure longer than 20 weeks can alter several S100 calcium-binding proteins—and those changes are linked to cancerogenesis [100]. Al can interfere with estrogen’s molecular mechanisms by disrupting estrogen binding to estrogen receptors (ER), the binding ability of ligand-receptor complexes to estrogen response elements (ERE) in DNA, and the transactivation of gene expression. This interference affects the cellular profiles of estrogen-regulated genes essential for normal cell growth control. MCF-7 cells demonstrate resilience to relatively high levels of Al exposure, and Al can increase estrogen-regulated reporter gene expression both in the absence and presence of E2. Given the widespread use of antiperspirants containing aluminum salts (resulting in long-term, low-dose exposure) and their interference with estrogen mechanisms, they likely play a role in breast cancer development [98].

### 3.4. Phenols

Bisphenol-A (BPA) [4,40-dihydroxy-2,2-diphenylpropane, CAS 80-05-7] is an industrial chemical obtained by synthesizing two phenolic groups with one acetone molecule. From 1940, this substance was used as a monomer for the production of polymers such as polycarbonates and epoxy resins, participated in the process of completing the polymerization of polyvinyl chloride and was used in the synthesis of flame-retardant tetrabromobisphenol-A [101,102]. Polycarbonates created with BPA are used in food containers and other products that come into contact with it, such as plastic plates, cups, microwave dishes, reusable bottles, and feeding bottles [102,103]. Epoxy resins are used to produce the inner coating of food and beverage cans. In addition, BPA is found in many other products, such as building materials, CD-ROMs, sunglasses, medical equipment, dental materials, and thermal paper [102,103,104,105]. Many studies have demonstrated the estrogenic properties of BPA [106] In addition, also bisphenol-F (bis (4-hydroxyphenyl) -methane, BPF) and bisphenol-S (4,4-dihydroxy-phenyl sulfone, BPS), which are analogs of BPA used in industry, exhibit estrogenic features [107]. BPF is contained in a small number of varnishes, adhesives, carpets, plastics, pipes, and food packaging, while BPS is often used as a component of phenolic resins and electroplating solvents [108]. Another alternative to BPA is BPAF, but recently, this substance has also been included in the EDC, showing its ability to bind to ERs, such as ER-alpha and ER-beta [108,109]. Moreover, BPAF may have stronger estrogenic activity than BPA, especially in breast cancer cells [109]. In 2006, EFSA published for the first time a BPA risk assessment based on a tolerable daily intake (TDI) of 50 μg/kg BW/day and concluded that human exposure via food does not exceed this value, even for infants and young children [91]. The ingesting of BPA in 2015 was approximately 7.7 million tons, and it is estimated that the annual consumption of this chemical substance will increase by another 3.1 million tons by 2022 [110]. BPA and its derivatives do not occur naturally in the environment; however, due to their enormous use in industry, pollution has increased and is now detected in the air, soil, water, dust, sediments, and human tissues [111,112]. The main source of bisphenols (BPs) in the environment is wastewater [102]. It has been shown that BPA and other BPs are not completely removed from wastewater (removal efficiency 62.5–99.6%) before being released into the environment [113].

Indoor dust is one of the sources of potential human exposure to bisphenols (BP). BPA can then enter the human body through inhalation and the contact of dust with the skin [114]. Children are most exposed to this source of BPA due to more frequent hand-to-mouth contact and, consequently, greater intake of dust than adults [27,114]. BPA was detected in dust from different rooms in a wide range of up to 10,000 ng/g of dust, and the median concentration obtained from dust by numerous groups ranged from 422 to 1460 ng/g in the US [91,97,101]. In a study by Cabana and Stepnowski, the concentration of BPA was assessed in various microenvironments in Poland, such as laboratories, offices, homes, and clothing stores. From the samples collected in this microenvironment, the highest BPA contamination was found in the laboratory dust (9504 ± 465 ng/g), which was related to epoxy resin floors [28]. Bisphenol-contaminated areas also include some river waters, groundwater, stream waters, estuaries, and lagoons [29]. Recently reported BPA levels in superficial soil samples from informal electronic waste recycling facilities and municipal open landfills in Indian cities ranged from 41 up to 459 ng/g [115].

Conversely, studies of river sediments from the Gulf of Gdańsk showed a maximum BPA concentration of 21.25 ng/g dry weight [116]. Analysis of 13 thermal printing papers showed the presence of BPA in eleven of them at a concentration of 8–17 g/kg. It has been shown that holding a receipt made of such paper for 5 s to the middle and index fingers transfers about 1 µg of BPA (0.2–6 µg) if the skin is dry and about 10 times more if the fingers are wet or very greasy. The exposure of a person who repeatedly touches paper for a thermal printer during 10 h of work can, therefore, amount to 71 μg/day. It is 42 times less than the current tolerable daily intake (TDI). Various factors, such as the greater contact surface of the paper with the body or using hand cream, can increase skin permeability and reduce the margin [104,117]. BPA was also found in socks for babies and toddlers in Spain (35.6% of samples exceeded the European Union (EU) standards of 0.1 ppm for toys) and in childcare articles and jewelry purchased from Israeli and international retail chains cheap online stores (17% of samples exceeded the EU migration norm) [118,119].

Many studies have demonstrated the estrogenic properties of BPA [106]. There exists a robust correlation between its presence and an elevated risk of breast, prostate, and uterine cancer [120]. Consequently, numerous environmental advocacy organizations recommend public avoidance of products containing BPA [16]. In addition, bisphenol-F (bis (4-hydroxyphenyl) -methane, BPF) and bisphenol-S (4,4-dihydroxy-phenyl sulfone, BPS), which are analogs of BPA used in industry, also exhibit estrogenic features [107]. Another alternative to BPA is bisphenol AF (BPAF), but recently, this substance has also been included in the EDC, showing its ability to bind to estrogen receptors, such as ERα, ERβ, and GPER [108,109]. BPA is a relatively weak environmental estrogen with an affinity for estrogen receptors (ERs) that is 1000–10,000 times lower than that of E2. It can bind to ERs located both in the plasma membrane and cytoplasm, thereby activating non-genomic effects of signal transduction as well as genomic pathways [121]. Gould et al. proved that BPA defines a new class of ERα-interactive compounds: being not merely an estrogen mimic but also producing its spectrum of activity via interacting with the ERα. Thus, BPA, a partial estrogen agonist, induces a conformation of activated ERα, which differs from other well-known ER ligands. Furthermore, it was also proven that BPA activates the ERα through a ligand-dependent mechanism [122].

### 3.5. Mycoestrogenes

Mycoestrogens are estrogen-like compounds produced primarily by mold fungi. Agricultural products, including grains and animal feeds, are susceptible to contamination by these mycotoxins. The most common mycoestrogens include zearalenone (ZAE) and ochratoxin A. These crops are exposed to contamination due to the ubiquitous presence of fungi, with moldy foods being the primary sources of these toxins. Environmental pollution further exacerbates the risk of fungal contamination.

#### 3.5.1. Zearalenone

Zearalenone, a mycoestrogen produced by various species of *Fusarium*, is notably resistant to environmental changes and heat treatment, which contributes to its stability during food storage and processing. It is commonly found in grains such as corn, wheat, rice, soybeans, and oats [123]. After ingestion by mammals, ZAE undergoes structural modifications, converting into alpha- and beta-zearalenone, both of which possess estrogenic structures and exhibit estrogenic activity. These metabolites compete with 17-beta-estradiol for binding to ERs. Zearalenone is highly toxic and can disrupt the reproductive system, liver and renal function, and immune response, and may influence carcinogenesis. Its estrogen-like structure enables zearalenone to interfere with physiological metabolic responses, leading to reproductive disorders via several pathways: (1) direct damage of germ cells and organs by binding to the ER, (2) increase the oxidative stress, (3) promote cell apoptosis due to DNA damage. Additionally, zearalenone triggers inflammatory responses and leads to hormonal secretion disorders. It has been shown to affect liver enzyme activity, and its ability to cause DNA damage can lead to hepatocyte injury, potentially resulting in liver cancer [124]. Zearalenone is also implicated in the carcinogenic processes of breast, prostate, and esophageal cancers by promoting apoptosis, generating reactive oxygen species (ROS), and causing DNA damage [123].

#### 3.5.2. Ochratoxin A

Ochratoxin A (OTA) is a significant mycotoxin produced by various species of *Aspergillus* and *Penicillium* fungi. Similar to zearalenone, OTA is widely distributed across food products, particularly cereals, and their derivatives, such as flour and bread [125]. The concentration of OTA in wheat and oats can range from 30 to 27,000 ng/g, with estrogenic agonistic effects observed [126]. While OTA does not appear to interact directly with ERs in the manner observed with zearalenone, it has been implicated in the disruption of steroid and hormone synthesis, and detailed pathomechanism requires further research [126]. *Aspergillus* species thrive in elevated temperatures and produce spores that are resistant to UV light, contributing to the presence of OTA in sunlight-exposed fruits and vegetables, including grapes. Notably, OTA has also been isolated from beverages such as coffee, tea, and wine, with average concentrations ranging from 0.1 to 100 ng/g. Furthermore, OTA can be transmitted through the food chain when animals consume grains contaminated with this mycotoxin, resulting in the contamination of animal-derived food products, including pork. Numerous animal studies provide substantial evidence of the carcinogenic properties of OTA. The International Agency for Research on Cancer (IARC) currently classifies OTA as possibly carcinogenic to humans (Group 2B), with a significant likelihood of reclassification to a known carcinogen (Group 1). Nevertheless, OTA poses considerable health risks to humans. Individuals exposed to OTA are at heightened risk for developing nephropathy due to its direct genotoxic effects and the induction of oxidative stress [127]. Table 1 below provides a comparison of the estrogenic and anti-estrogenic activities exhibited by various xenoestrogens.

## 4. The Influence of XEs Exposure on Cancer Development

**Figure 2:**
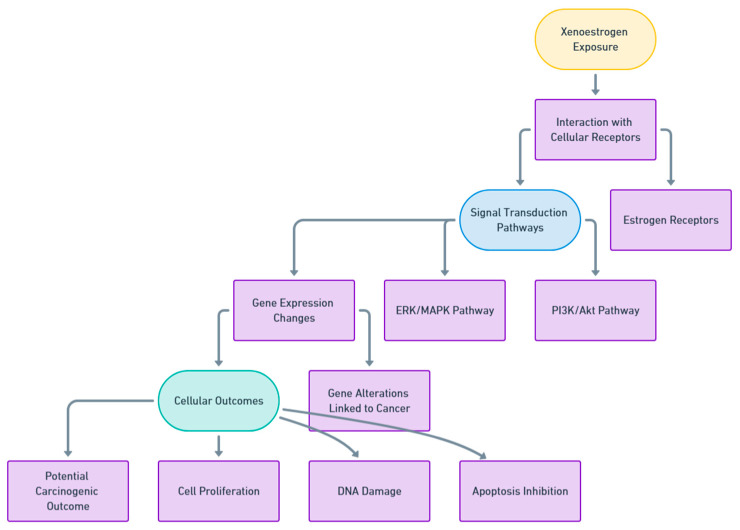
A schematic diagram illustrating the molecular pathway by which XEs exposure may lead to carcinogenesis. The diagram shows the interaction of xenoestrogens with cellular receptors, activation of signal transduction pathways, subsequent gene expression changes, and cellular outcomes, which collectively contribute to potential cancer development (prepared by using https://whimsical.com/, accessed on 6 November 2024).

XEs are well known to be associated with various types of cancer genesis. Genomic and nongenomic pathways activated by XEs ligands control each other [133]. The results of this combination may include proliferative diseases of breast and prostate tissues [134]. Frequent exposure to XEs present during the perinatal period initiates a process termed epigenome reprogramming. It was proven that perinatal exposure to EDCs, such as BPA, genistin, and diethylstilbestrol, increase the risk of embryonal cancer development by inducing angiogenic ER signaling and activating phosphoinositide-3-kinase/protein kinase B pathway (PI3K/AKT) [135,136]. The epigenome disruption occurs due to AKT phosphorylation and histone methyltransferase EZH2 inactivation. The long-time accumulation of XEs from multiple exposure sources leads to total intake that exhibits a “cocktail mixture effect,” resulting in biological impacts on human physiology. Figure 2 demonstrates the proposed molecular pathway by which XEs exposure affects cellular signaling and gene expression, potentially leading to carcinogenic outcomes.

These effects present a lifelong risk, especially in older years when the levels of endogenous estrogens drop significantly, and the total intake amount of XEs is at its highest. The specific effects of substances differ based on their exposure concentrations. For instance, some substances (e.g., tamoxifen, genistein) display a mixed agonist/antagonist effect, where lower concentrations often result in a stimulatory effect on cell proliferation, and higher concentrations exert opposite effects [137]. Both genomic and non-genomic cellular reactions to XEs need to be investigated in order to create a large database. The exact effects of this cocktail mixture are difficult to examine, not only due to different mechanisms of actions of the ingredients but also due to complex interactions between them. All those mechanisms have to be taken into consideration when assessing functional outcomes [138]. Different models, such as the concentration addition (CA) model or the independent reaction (IA) model, have been proposed to measure and describe the effects of XEs. The CA model presents the cumulative effects of XEs. Other models, such as the model of generic response addition (GRA) have been proposed in order to account for specific features such as maximum response, sensitivity, and potency [139].

### 4.1. Breast Cancer

Due to the various locations of ERs in human organs, there are different types of mechanisms in which estrogens stimulate cancer growth. Probably the most discussed cancer caused by exogenous estrogens is breast cancer (BC). The percentage of diagnosed women has risen progressively since the early 20th century. According to the GLOBOCAN database, in 2020, almost 2.3 million new female breast cancer cases were diagnosed [140]. Several studies show that BC is more often diagnosed in developed countries [87,141]. Exposure to XEs may have similar effects as exposure to natural E2 and, therefore, may be responsible for at least some cases of BC. The total lifetime accumulation of XEs could explain why so many women over the age of 50 are diagnosed with this carcinoma [142]. The main sources of exogenous estrogens are combined oral contraceptives (COCs) and hormone replacement therapy (HRT). The conclusive risk of BC development is derived solely from the cumulative impact of various sources utilized at varying frequencies, in conjunction with established and well-recognized risk factors, including advanced age, familial history, and other contributing factors [143].

There are two of the most frequently discussed molecular pathways of BC advancement in the available literature. One, initiated by activation of ER, results in the expression of genes, causing rapid proliferation of cells. Importantly, BRCA1, a caretaker tumor suppressor gene, controls the proliferation of cells stimulated by estrogen. Levels of BRCA1 expression, as well as other tumor suppressors such as p53, stay in close interaction with levels of estrogen. Typically, high estrogen levels are associated with high levels of BRCA1 expression and tumor suppressor activity. Inherited or acquired mutation and downregulation of the gene results in impaired ability to repair genes; therefore, elevated levels of estrogens could cause transformation, excessive proliferation, and cancerous changes. Other studies show that BPA (Bisphenol-A) stimulates the up-regulation of BRCA1, BRCA2, and BRCA51 and the expression of BRCA1 and BRCA3 in MCF-10F non-tumorigenic cells, genes responsible for DNA repair. This suggests that BPA could increase mutation frequency in BRCA1 carriers. It is also concluded that BPA induces methylation changes in human breast epithelial cells. Cells deficient in the BRCA1 gene are more sensitive to BPA [144]. According to the study by Kang et al. [145], reactive oxygen species (ROS) imply high DNA damage levels in BRCA1-deficient cells. PCB, Cd, and BPA have not induced levels of ROS significantly, in contrast to paraquat, TCDD (herbicide), 2-OH-E2, and 4-OH-E2 (catechol estrogens). Considering the results described above, the studies revealed that concurrent exposure to some xenobiotics increases the risk of breast cancer carrying BRCA1 deficiency [145]. The second pathway of breast cancer development includes oxidative metabolic products of estrogenic substances [33]. Natural E2 is metabolized through two pathways: the first one yields 2-hydroxy estrone, and the second yields 16-hydroxy estrone. Metabolizing E2 into 2-hydroxy estrone, which is estrogen, is less damaging to tissues. It is also the case that 16- hydroxy estrone is present in high concentrations in breast cancer tissues and has DNA-damaging properties. XEs such as DDT, DDE, atrazine, and kepone promote the 16-hydroxy estrone pathway, thereby increasing the risk of BC [146]. BPA, BBP (benzyl butyl phthalate), DDT, and DDE (dichloro-diphenyl-dichloroethylene), are thought to stimulate BC development. BPA mimics E2 and competes with it for binding to ERα and ERβ. Although the affinity of BPA is at least ten thousand times lower than that of E2 for ERα and ERβ, BPA may stimulate the neoplastic transformation of breast epithelial cells [147,148]. BBP, as an ER agonist, has the potential to initiate BC formation [147]. Both BBP and BPA are potentially carcinogenic, but only in high concentrations. There are many indications that elevated levels of DDE are involved in breast cancer in women. In a hematological investigation conducted by Wolff et al. [149], it was observed that women exhibiting markedly elevated levels of DDE had a fourfold higher risk of developing breast cancer compared to individuals with lower DDE concentrations [149]. Other studies have reported raised levels of DDE, BBP, and polybrominated biphenyls in BC patients [148]. Contrarily, in 1997, it was observed that current data do not support the role of PCBs and DDE as etiological factors [149]. Exposure to DES (diethylstilbestrol, a synthetic estrogen) and BPA in utero have been shown to enhance zest homolog 2 (ZH2) expression. The study conducted by Duan et al. revealed that the enhancer of zest homolog 2 (EZH2) promotes the formation of cytoplasmic chromatin fragments (CCF) in BC cells [150]. DES and BPA form a potential mechanism for increased BC risk [151,152]. A modest elevation in the occurrence of breast carcinoma has been documented among individuals utilizing combined oral contraceptives (OCs) and hormone replacement therapy (HRT) [153].

Estrogens act on the uterus, i.e., endometrial cancer progression may be estrogens dependent [154]. In hormonal imbalances caused by excess estrogenic substances (XEs), progesterone will not be able to compensate for estrogen effects. Therefore, abnormalities in DNA replication and carcinogenesis may occur. Zearalenone (ZAE), present in contaminated corn cobs, rice, cereals, wheat, and beer, undergoes rapid metabolism upon ingestion. Despite its swift metabolic transformation, ZAE and its metabolites can accumulate in reproductive organs, exhibit estrogenic properties attributable to structural similarities, and exert influence on estrogen receptors [155]. It is hypothesized that ZAE and its metabolites can induce tumor cell proliferation in the uterus. One of the studies examined estrogenic compounds in the induction of cell proliferation; however, only the combination of XAN:ZEN:alpha-ZEL (XAN- xanthohumol) has been found to increase proliferation significantly [154]. Nowadays, COCs are reported to reduce the risk of both ovarian and endometrial cancer [153].

ERα is also expressed in male mammary glands, where low BPA exposure is associated with increased proliferation and apoptosis. BPA modifies the morphology of the male mammary gland in adulthood and leads to gynecomastia, the most common male breast condition that affects approximately 50% of boys at sexual maturity [156]. Although changes in male mammary gland morphology vary with the level of BPA exposure, it has been reported that at any dose, BPA changes male mammary gland development [22]. In a study by Clemons et al. [157], low-dose DES treatment of castrate-resistant prostate cancer (CRPC) resulted in gynecomastia in 59% of patients [157]. There is no evidence of an association between gynecomastia and breast cancer. However, exposure to exogenous estrogens is known to increase the risk of BC in men. Mutations in the BRCA1 and BRCA2 genes are a risk factor for breast cancer in men and may be a consequence of exposure to XEs [158]. Conversely, studies have demonstrated that soy isoflavones contribute to the suppression of breast cancer cell proliferation via ER-independent mechanisms, specifically by inhibiting DNA topoisomerases and tyrosine kinases, as suggested by certain investigations [159,160,161]. It was noted that the correlation between soy consumption and breast cancer does not focus on the quantity of consumption itself. Friz et al. sought to determine whether the amount of soy and isoflavones ingested has a positive influence on pre- and post-menopausal women. The authors concluded that soy isoflavone intake may decrease the risk of BC in both pre-menopausal and post-menopausal women [159].

### 4.2. Prostate Cancer (PC)

Estrogens interact with normal prostate tissues, impacting their growth, differentiation, and homeostasis [6,14]. Epidemiological studies concluded that African American men whose levels of estradiol in serum are higher carry a bigger risk of developing prostate cancer (PC) [162]. Among the elderly, testosterone levels drop while estradiol levels remain constant [163]. The prostate is exposed to changes in the ratio of sex hormones. During those fluctuations, androgen levels drop, causing atrophy of prostate tissues. The rebuilding results in the regrowth of prostate epithelium. Regrowth enables the clonal multiplication of cells containing oncogenic mutations. That suggests a connection between the estradiol/testosterone ratio and the probability of PC development. The prostate is exposed to changes in the ratio of sex hormones [164]. However, some studies give up this idea, concluding that there is no clear evidence of the strong correlation between the development of PC and estrogen/androgen ratio or elevated estrogen levels [14,165]. It was also reported that induction of metaplastic changes in the prostate does not occur, even though estrogen and androgen act synergistically [31,149]. Some studies suggest that dietary recommendations should be made for patients with prostate cancer and call for the development of ER-specific treatments to shrink tumors or slow tumor progression. These conclusions have been drawn from proving that some compounds, such as phytoestrogens, coumestrol, and genistein related to soy, have profound stimulatory effects on prostate cancer cell numbers. It has also been demonstrated that exposure to resveratrol and BPA may lead to similar risks. Exposure advice may need to be differentiated depending on the PC stage, considering that BPA and genistein affect the number of viable cancer cells differently [166].

The development of most recurrent PCs is driven by inappropriate androgen receptor activation. It has been reported that estrogenic environmental compounds such as BPA influence androgen receptor (AR) activity and subsequent progression to androgen independence. BPA at low, environmentally relevant doses can enhance the transcriptional efficacy of androgen receptor PC cells. It also activates multiple tumor-derived mutant AR alleles and moderately induces wild-type AR activity in prostate cancer cells. Exposure to high BPA doses separates androgen-mediated transcription from AR-mediated mitogenesis in prostate carcinoma cells [167]. Phytoestrogen was hypothesized to at least partially affect PCs epidemiology. It was proven in studies [83,168] that phytoestrogens coming from dietary income are connected to PCs incidence. Few tests performed on humans suggest mild protection from cancerous changes [169,170], but more studies should be performed to discover the full impact of phytoestrogens on humans [146]. Phytoestrogens are reported to be consumed in many products. In countries such as Japan, diet contains a lot more phytoestrogens, which could partially explain the disproportion in rates of breast, colon, and prostate cancer deaths. A diet rich in phytoestrogen isoflavonoids (alongside lignin and fiber) is beneficial in terms of the development of cancer [149].

### 4.3. Other Carcinomas: Uterine Cancer, Testicular Cancer, Ovarian Cancer, Lung Cancer

XEs are linked to cancers of different organs. There are implications that XEs might affect the development of renal cancer as well as pancreatic cancer [171]. In pancreatic cancer cells, ERα and ERβ receptors are modulated by estrogens. Substances with XEs’ properties, such as benzo(a)pyrene and other contaminants found in food, are reported to increase the risk of BC. In addition, methylnitrosamino-pyridyl-butanone (a tobacco carcinogen) has been found to modulate ER pathways in this cancer, as well as in lung adenocarcinomas [172,173]. The ERβ-related compound is reported to increase the risk of lung cancer [174]. Other substances that are linked to lung cancer and estrogen receptors are cotinine, polonium 208 (which might have similar properties to metal estrogens), PAHs (a class of substances found in mixtures of agents in air pollution), arsenic, dioxins, and heavy metals (from industrial emissions). Some of them demonstrate properties similar to XEs, and some affect estrogenic homeostasis or ER receptors. Transplacental exposure to XEs due to maternal tobacco smoking to XEs might be related to an increased risk of BC [175]. Testicular cancer is hypothesized to be linked to XEs, especially due to its unexplained increase in incidence in the last decades. Epidemiological studies suggest that this might be explained by prenatal exposure to XEs [176]. Other hypotheses are being formulated and checked as further research is necessary to follow.

Sawicka et al. [177] investigated the role of 17β-estradiol and its metabolites: 2-MeOE2, and 16α-OHE1 in exposure to the metalloestrogen Cd on ovarian cancer cells in vitro [177]. Given the prevalent occurrence of multidrug resistance (MDR) in ovarian cancer, an evaluation of the impact of estrogens and Cd on the MDR phenomenon in SKOV-3 cells was conducted by the authors through measurements of P-glycoprotein (P-gp) expression levels. E2 and Cd exhibited an augmentation in P-gp expression in SKOV-3 cells, whereas 2-MeOE2 demonstrated a reduction in P-gp expression, suggesting a potentially advantageous effect for the prevention of MDR.

In the last few years, benzophenone-1 (BP-1) has fallen into the category of emerging concern as a contaminant associated with personal care products, and recent reports have highlighted its capability to induce xenoestrogenic effects [178]. Liu et al. examined the disruptive effects of Benzophenone-1 (BP-1) at concentrations environmentally relevant (10-9–10-6 M) on ER α-associated signaling pathways [179]. The authors revealed that the activation of estrogen receptor ERα by BP-1 initiates crosstalk between ERα and the Wnt/β-catenin pathway, resulting in aberrant stimulation and progression of SKOV-3 cancer cells. Table 2 provides a summary of the effects of the most prevalent xenoestrogens encountered in everyday life, along with the primary sources of these substances.

## 5. Conclusions

The diverse and extensive applications of XEs necessitate a comprehensive understanding of their impacts on human health. Given their widespread presence in consumer products and the environment, a systematic evaluation of XEs exposure is critical, particularly due to the disruptive and often harmful effects on biological systems. The current body of research demonstrates a significant correlation between XEs exposure and the progression of various cancers, including breast, bladder, renal, ovarian, and prostate cancers. Beyond oncological implications, XEs are also implicated in disrupting the normal function of critical organs, notably the lungs, kidneys, and uterus. This wide-ranging impact reinforces the urgent need for more precise regulatory measures and public health strategies.

Although the complete elimination of XEs from daily life may be unfeasible, exposure can be significantly reduced through informed choices. Strategies such as selecting food with minimal packaging contamination, using alternatives to traditional antiperspirants, and choosing products free from harmful chemicals can help mitigate risk.

## Figures and Tables

**Table 1:** Comparison of the estrogenic and anti-estrogenic activity of xenoestrogens.

Xenoestrogen	Effect of Action	References
***Phytoestrogenes***		
Genistein (7,4′-dihydroxy-6-methoxyisoflavone)	Estrogenic and anti-estrogenic activity, Inhibition of 5α-reductase activity, Inhibition of aromatase activity	[4,128]
Daidzein (7,4′-dihydroxy isoflavone)		
Biochanin A (5,7 dihydroxy-4′-methoxy isoflavone)		
Formononetin (7-hydroxy-4′-methoxyisoflavone)		
Equol		
***Pesticides***		
Thiacloprid	Estrogenic activity at high concentrations	[129]
Imidacloprid	Estrogenic activity at high concentrations	[129]
Glyphosate	Inhibition of aromatase activity (up to 30%)	[129]
Metiokarb	Estrogenic activity at high concentrations	[130]
***Metaloestrogens***		
Cadmium	Estrogenic activity	[131]
Aluminium	Interference with estrogen binding to the receptor and estrogen-dependent reporter gene expression	[98]
***Phenols***		
Bisphenol A	Primarily estrogenic and anti-androgenic effect, Anti-estrogenic and androgenic effect also described, Similar hormonal activity	[108]
Bisphenol F		
Bisphenol S		
Bisphenol AF		
***Mycoestrogenes***		
Zearalenone	Estrogenic and anti-androgenic effect	[123,124,132]
Ochratoxin A	No direct effect on estrogen receptors, Disruption of steroid synthesis, Exact mechanism unknown	[126]

**Table 2:** Summary of the effects of the most prevalent xenoestrogens encountered in everyday life on the body and presentation of the sources of these substances.

Xenoestrogen	Source	Health	References
Cd	Sources of Pollution from Industrial and Agricultural Practices: Consumption of contaminated water and/or food, esp. mussels, crustaceans, rice Inhalation of cigarette smoke Environmental impact: cadmium (Cd) does not degrade in the environment, leading to constantly increasing exposure and contamination	Increased risk of breast, bladder, renal, and ovarian cancer	[73,85,89,180,181]
Al	Food: legumes, nuts, oilseeds, spices. Cosmetics: antiperspirants	Stimulate breast cancer development	[94,98,182]
Isoflavones: genistein, glycytin, biochanin A, formononetin,	Food: legumes (Fabaceae family), soy products, chickpeas, nuts, red and white clover, alfalfa	Profound stimulatory effects on prostate cancer cell proliferation. In contrast to lowering the risk of breast cancer	[34,159,166,183]
Resveratrol	Food: grapes, wine	Prevention of cardiovascular diseases. Potential use in cancer prevention and therapy	[4,46,47]
Bisphenol-A	Food containers and packaging: plastic plates, cups, microwave dishes, reusable bottles Building materials and miscellaneous products: CD-ROMs, sunglasses, medical equipment, dental materials, thermal paper, varnishes, adhesives, carpets, plastic, pipes, wastewater, dust	BPA increases mutation frequency in BRCA1 carriers, and high concentration can lead to breast cancer.	[103,114,144]
Zearalenone	Food: grains: corn, wheat, rice, soybeans, oats	Reproductive disorder, hepatotoxicity, nephrotoxicity. Promotion of carcinogenic process of breast, prostate, esophageal, and liver cancer.	[123,124]
Ochratoxin A	Food: cereals, wheat, oats, grapes, coffee, tea, wine, meat.	Nephrotoxicity, according to IARC group 2B (possibly cancerogenic effects)	[125,126,127,184]

## Data Availability

Data is available upon request.
